# Evaluating the Long-Term Effectiveness of School-Based Depression, Anxiety, and Substance Use Prevention Into Young Adulthood: Protocol for the Climate School Combined Study

**DOI:** 10.2196/11372

**Published:** 2018-11-06

**Authors:** Louise Birrell, Nicola C Newton, Tim Slade, Catherine Chapman, Louise Mewton, Nyanda McBride, Leanne Hides, Mary Lou Chatterton, Steve Allsop, Annalise Healy, Marius Mather, Catherine Quinn, Cathrine Mihalopoulos, Maree Teesson

**Affiliations:** 1 National Health and Medical Research Council Centre of Research Excellence in Mental Health and Substance Use National Drug & Alcohol Research Centre University of New South Wales Sydney Australia; 2 National Drug Research Institute Curtin University Perth Australia; 3 Centre for Substance Abuse Research School of Psychology University of Queensland Brisbane Australia; 4 Population Health Strategic Research Centre Deakin University Geelong Australia

**Keywords:** alcohol abuse, prevention, depression, anxiety, costs and cost analysis, school, eHealth

## Abstract

**Background:**

Mental health and substance use disorders are the leading causes of global disability in children and youth. Both tend to first onset or escalate in adolescence and young adulthood, calling for effective prevention during this time. The Climate Schools Combined (CSC) study was the first trial of a Web-based combined universal approach, delivered through school classes, to prevent both mental health and substance use problems in adolescence. There is also limited evidence for the cost-effectiveness of school-based prevention programs.

**Objective:**

The aim of this protocol paper is to describe the CSC follow-up study, which aims to determine the long-term efficacy and cost-effectiveness of the CSC prevention program for depression, anxiety, and substance use (alcohol and cannabis use) up to 7 years post intervention.

**Methods:**

A cluster randomized controlled trial (the CSC study) was conducted with 6386 participants aged approximately 13.5 years at baseline from 2014 to 2016. Participating schools were randomized to 1 of 4 conditions: (1) control (health education as usual), (2) *Climate Substance Use* (universal substance use prevention), (3) *Climate Mental Health* (universal mental health prevention), or (4) *CSC* (universal substance use and mental health prevention). It was hypothesized that the CSC program would be more effective than conditions (1) to (3) in reducing alcohol and cannabis use (and related harms), anxiety, and depression symptoms as well as increasing knowledge related to alcohol, cannabis, anxiety, and depression. This long-term study will invite follow-up participants to complete 3 additional Web-based assessments at approximately 5, 6, and 7 years post baseline using multiple sources of locator information already provided to the research team. The primary outcomes include alcohol and cannabis use (and related harms) and mental health symptoms. An economic evaluation of the program will also be conducted using both data linkage as well as self-report resource use and quality of life measures. Secondary outcomes include self-efficacy, social networks, peer substance use, emotion regulation, and perfectionism. Analyses will be conducted using multilevel mixed-effects models within an intention-to-treat framework.

**Results:**

The CSC long-term follow-up study is funded from 2018 to 2022 by the Australian National Health and Medical Research Council (APP1143555). The first follow-up wave commences in August 2018, and the results are expected to be submitted for publication in 2022.

**Conclusions:**

This is the first study to provide a long-term evaluation of combined universal substance use and mental health prevention up to 7 years post intervention. Evidence of sustained benefits into early adulthood would provide a scalable, easy-to-implement prevention strategy with the potential for widespread dissemination to reduce the considerable harms, burden of disease, injury, and social costs associated with youth substance use and mental disorders.

**International Registered Report Identifier (IRRID):**

PRR1-10.2196/11372

## Introduction

### Background

Mental health and substance use disorders are the leading causes of global disability, accounting for 25% of total disability in children and youth [1]. Every year, mental and substance use disorders conservatively cost the Australian community over Aus $12.7 billion [2]. The burden of substance use and mental disorders now account for 1 in every 10 lost years of health globally [3]. The most common mental disorders are anxiety and depression [4,5], with the most commonly used substances in Western countries, such as Australia, being alcohol and cannabis. To reduce the cost and burden of depression, anxiety, and substance use, timely and effective prevention is critical.

Epidemiological studies show that between 40% [6] and 50% [7] of the population in Western countries will suffer from a depressive, anxiety, or substance use disorder during their lifetime. Furthermore, depression, anxiety, and substance use disorders typically emerge before the age of 25 years [8]. From the ages of 13 to 24 years, there is an increased susceptibility for the development of depression, anxiety, and substance use disorders. Longitudinal life course studies show that transitions from childhood to adolescence and from adolescence to young adulthood are marked by significant increases in anxiety disorders, depression, and substance use disorders [9]. In addition, even small elevations in mental health symptoms in adolescence increase the likelihood of developing a full-blown mental disorder later in life [10]. Substance use during adolescence is also a significant global problem, resulting in a number of adverse outcomes including violence, accidental injury, self-harm, suicide, and an increased risk of developing mental illness [11]. Although the majority of adolescents will not meet criteria for a full-blown substance use disorder [12], a substantial portion will use alcohol at harmful levels [13]. Data from nationally representative surveys consistently show rates of substance use (namely alcohol use) increase steeply between the ages of 13 and 18 years [14-16]. In the general population, approximately 25% of people with a substance use, anxiety, or mood disorder will experience comorbidity with another class of these disorders in any 12-month period [17]. Of particular concern, individuals with comorbid disorders are harder to treat, suffer a more chronic illness course, and experience poorer outcomes later in life than those with no disorder or single disorders [18,19].

To halt the escalation and associated burden of disease, prevention efforts need to be commenced before the onset and acceleration of substance use, depressive, and anxiety symptoms into well-established patterns and disorders. Adolescence is a key time to do this. School-based programs have been shown to reduce both substance use and depression and anxiety symptoms [20-22]. However, to date, prevention programs tend to target single disorders in isolation, ignoring the comorbidity and common risk factor shared by substance use and mental disorders [23,24]. Until recently, there were no prevention models targeting depression, anxiety, and substance use simultaneously.

Few studies have examined the effectiveness of prevention approaches for substance use, depression, and anxiety beyond secondary school. There is limited evidence from studies in the United States [25,26] that receiving the universal Life Skills Training substance use prevention program in year 6-7 (ages 12-13 years) reduced risk of alcohol-related problems and illicit drug use into early adulthood (ages 18-22 years). Further investigations of the Life Skills Training program have indicated that these reductions in substance use, in turn, demonstrated significant secondary benefits on depression symptoms at the age of 22 years [27]. According to recent reviews, the durability of universal prevention programs for anxiety and depression has not been investigated beyond 4 years post intervention and into the young adult years [28-30]. The secondary effects of prevention for anxiety and depression on substance use are rarely investigated and have been identified as a high priority future direction in prevention research [31]. Moreover, assessment over the longer term for interventions instigated in adolescence is important as adolescents are increasingly exposed to drugs and alcohol, and personality vulnerabilities are triggered by the unique challenges associated with early adulthood [32].

### The Climate School Combined Study: First Randomized Controlled Trial of Simultaneous Universal Prevention for Anxiety, Depression, and Substance Misuse

The Climate School Combined (CSC) study commenced in 2014 as the first randomized controlled trial (RCT) of a combined approach to preventing depression, anxiety, and substance misuse (focusing on alcohol and cannabis) in adolescence [33]. This study was a 4-armed, cluster RCT, in which participating schools were randomly allocated to 1 of 4 conditions: (1) *CSC intervention*; (2) *Climate Schools Substance Use*; (3) *Climate Schools Mental Health*, or (4) Control (health and physical education as usual). Participants allocated to the *CSC* intervention received 18 × 40-min classroom lessons focused on depression, anxiety, alcohol, and cannabis. Each lesson includes both computer-based and manualized classroom activities. The computer-based component is delivered on the Web to individual students who log on to view cartoon storylines that impart information about anxiety and depressive symptoms, alcohol, and cannabis. The classroom activities are delivered by the teacher and aim to reinforce the learning outcomes outlined in the cartoons and allow interactive communication between students. These lessons adopt a harm minimization approach in relation to substance use and utilize cognitive behavioral skills and strategies to assist students in identifying and reducing problematic mental health symptoms. Those allocated to *Climate Schools Substance Use* intervention received 12 × 40-min lessons focused on alcohol and cannabis use, those allocated to the *Climate Schools Mental Health* intervention received 6 × 40-min lessons focused on anxiety and depression, whereas those in the control condition received health education as usual. Further details about the intervention components and groups have been previously reported [33]. The primary aim of the original CSC study was to assess the effectiveness of delivering a comprehensive prevention strategy (the *CSC* intervention) targeting depression, anxiety, and substance use (alcohol and cannabis) in reducing the onset and escalation of mental health symptoms, substance use and related harms, and increasing knowledge in relation to these issues. A total of 71 schools and 6386 students aged 13 to 14 years at baseline participated in the trial. Although the initial phase of the study did not specifically aim to test the cost-effectiveness of the intervention, resource use questions used in cost-effectiveness analysis were included in the study from baseline (2014).

### Sustaining Prevention Effects Into Young Adulthood: The Need for Longer-Term Follow-Up

At ages 17 to 18 years, the CSC trial cohort is now nearing early adulthood in 2018. This transition, from adolescence to early adulthood, represents a unique developmental period characterized by numerous personal and social role changes including new social relationships and living arrangements, increased financial and social independence, and pursuit of employment and/or higher education. Along with increased exposure to alcohol and cannabis during this period, mental health symptoms often become more pronounced with the onset of new challenges, increased autonomy, and formation of new friendship circles. A review of longitudinal epidemiological studies focusing on the transition from adolescence to young adulthood found that rates of any mental or substance use disorder more than doubled, as did the use of illicit drugs [9]. Specifically, substance use, anxiety, and depression begin to increase in adolescence and continue to increase significantly into early adulthood [34-36].

Despite evidence demonstrating that school-based prevention efforts can interrupt the trajectory of growth in substance use and mental health symptoms during adolescence [21,22,37], very little research has focused on whether these effects can be sustained into the critical period of young adulthood [38]. The majority of existing prevention programs have a very limited evidence base beyond 3 years [22,38,39]. It is therefore unclear whether prevention effects are sustained into young adulthood, when adult vocational and social roles are established, and rates of substance use and mental health problems are highest. Even small disruptions have potentially significant economic consequences, with economic modeling suggesting that even modest long-term reductions in substance use would lead to substantial societal benefits [40]. Evaluation of long-term outcomes and cost-effectiveness of school-based prevention programs for substance use, anxiety, and depression is a critical knowledge gap, and the long-term effectiveness of combining mental health and substance use prevention is unknown.

### The Climate Schools Combined Long-Term Follow-Up Study

The CSC long-term follow-up study will be the first in the world to examine the long-term effectiveness of a combined approach to the universal prevention of anxiety, depression, and substance use disorders delivered on the Web. It will extend the follow-up of the existing CSC cohort by an additional 3 time points (5, 6, and 7 years post initial baseline assessment in 2014). There is limited evidence to suggest that when delivered in isolation, mental health and substance use prevention programs have secondary benefits on comorbid conditions [41,42]. This suggests the possibility of powerful multiplicative effects of mental health and substance use prevention when delivered in combination. This study will provide evidence on the long-term effectiveness of a combined approach to prevent mental health (anxiety and depression) and substance use (alcohol and cannabis) problems and evaluate the cost-effectiveness of such an approach. This combined approach will be compared with mental health and substance use prevention delivered in isolation and to education as usual (a control group). Primary outcomes include alcohol and cannabis use and related harms, and mental health symptoms. An economic evaluation of the program will utilize data linkage as well as self-reported resource use (use of health care staff time, facilities, and consumables) and quality of life measures. By investigating the long-term effects and cost-effectiveness of a combined approach to preventing mental health and substance use problems, this study will provide crucial information about which prevention approaches are most sustainable and whether and when additional booster sessions might be needed. To further understand intervention effects over the long term, it is important to explore potential moderators and mediators of the intervention. Research has shown that emotion regulation [43], self-efficacy [44], personality domains (such as perfectionism) [45], social networks [46], and broad internalizing and externalizing domains [47] are important factors to consider in relation to substance use and mental health and will be considered as potential moderators and/or mediators in this study.

### Cost-Effectiveness of Universal School-Based Prevention

There is limited evidence demonstrating the value for money of school-based programs to prevent depression, anxiety, and minimize substance abuse. An economic evaluation of school-based programs to prevent depression in adolescents aged 11 to 17 years demonstrated that both were cost-effective in an Australian context [48]. However, this was a modeled evaluation limited to the prevention of depression, based on a number of assumptions and used disability-adjusted life years as an outcome measure. A total of 4 cost-benefit analyses evaluated school-based programs to prevent substance use. The benefits of the prevention programs and the monetary benefits of reducing substance use over a lifetime outweighed the costs to deliver the programs in schools [49-51]. However, these were also model-based evaluations and conducted in the United States education and health systems. An economic evaluation conducted alongside an RCT of these interventions has not been undertaken nor has an economic evaluation of a combined Web-based approach to prevent mental and substance use disorders.

### Aim

The study will conduct a long-term (7-year) follow-up of the first RCT of a combined Web-based substance use and mental health prevention approach addressing the following research questions:

1. RQ1: Is the combined approach used in the CSC program more effective in the long-term across the transition into early adulthood (ages 18-21 years) compared with: (1) universal substance use prevention (*Climate Substance Use)*, (2) universal mental health prevention (*Climate Mental Health)*, and (3) education as usual (control condition) for:
  - reducing the use and harmful use of alcohol and cannabis
  - reducing overall symptom levels of anxiety and depression
2. RQ2: How cost-effective is a combined approach to prevention over the long-term?

We hypothesize that the combined prevention model (CSC program) will be cost-effective compared with (1) school-based prevention as usual, (2) stand-alone universal school-based substance use prevention, and (3) stand-alone anxiety and depression prevention, where Aus $50,000 per quality-adjusted life year is taken as the benchmark for cost-effectiveness in Australia.

## Methods

### Ethics Approval and Consent to Participate

The study was approved by the University of Sydney Human Research Ethics Committee, Australia (2018/906), and all participants provided informed consent to participate in the original CSC study. All participants will provide additional informed consent before participating in further follow-up surveys.

### Study Design

This trial is registered with the Australian and New Zealand Clinical Trials Registry: 12613000723785. A total of 88 schools from the Australian states of New South Wales, Western Australia, and Queensland were recruited to the CSC trial in 2014. A total of 17 schools withdrew after randomization (primarily due to time constraints). The final cohort at baseline consisted of 6386 year 8 students from 71 schools (mean age 13.5 years [SD 0.6], 54.84% [3502/6386] female, 81.24% [5188/6386] born in Australia). Participating schools were randomized to 1 of 4 conditions: (1) Control (health education as usual), (2) *Climate Substance Use* (universal substance use prevention), (3) *Climate Mental Health* (universal mental health prevention), or (4) *CSC* (universal substance use and mental health prevention). Blocked randomization was used, allocating schools to the 4 conditions in equal ratios in blocks of 4. The CONSORT diagram (see Multimedia Appendix 1) summarizes participant flow and retention rates through the study for each condition. Comprehensive information about the intervention content, delivery, and study design of the original CSC study has been published in the original CSC study protocol [33]. The completed CSC study assessments and timeline for extended follow-up assessments can be seen in Multimedia Appendix 2.

### Procedure

The CSC long-term follow-up study will extend data collection up to 7 years post baseline. Using multiple sources of locator information already provided to the research team (eg, email addresses, address, phone number, and Facebook usernames), all participants will be invited to consent to take part in the long-term follow-up and then complete 3 Web-based assessments at approximately 5, 6, and 7 years post baseline. Participants in the state of Queensland complete school 1 year earlier than participants in New South Wales and Western Australia. To collect data from Queensland participants in their first year post school, follow-up will commence in Queensland from August 2018 to January 2019, whereas data collection will run from January 2019 to June 2019 in New South Wales and Western Australia. Participants will consent to take part in the longitudinal follow-up study and provide additional consent to release their Medicare Benefits Schedule and Pharmaceutical Benefits Scheme information to the research team. Subsequent contact with students will be made via email invitation or via school with reminder emails and texts sent once a week for 3 weeks. Those who cannot be reached via email will be contacted via alternative forms of locator information, including short messaging service (SMS) and social media. If no response is received, participants will be followed up via phone calls, and paper surveys will be mailed to their home address. Participants will be contacted via the locator information provided until a response is received.

“Participants will be directed to the CSC website through a personalized URL to complete written consent procedures and complete the survey (approximately 30-45 min in duration) on the Web. Responses will be deidentified and linked over time using a unique identification code. Participants will be reimbursed Aus $20 in the form of a gift voucher for each survey occasion they complete. A duty of care procedure has been developed and approved by the University of New South Wales Human Research Ethics Committee and will be followed if a participant self-identifies as at risk of harm during the study. This includes automatic emails to participants with detailed information about support services if their response indicates they are at risk of harm.

### Sample Size Calculations

Participants for this study come from 6386 students from 71 schools recruited to the original CSC study. Power calculations for the original trial were based on methods developed to detect intervention by time interactions in longitudinal cluster RCTs [52] and ensured adequate power to detect clinically significant differences across groups both in the total sample *and* in each of the 3 states of Australia where recruitment took place. These calculations accounted for 10% dropout at the school level and indicated that 2800 students recruited from 28 schools in each state (for a total of 8400 students) would achieve 80% power to detect a between-group mean difference of 0.15 (at the *P*<.05 level) with 7 measurement occasions. In our original study, we achieved a total sample size of 6386 students. Although this is not sufficient to do analyses at the state level, the total sample size is more than sufficient and far surpasses the 2800 required to detect the expected differences across the whole sample. As initiation and frequency of substance use as well as levels of depression and anxiety increase over the transition to early adulthood [9], larger effect sizes are expected over the longer-term follow-up. Thus, even allowing conservatively for dropout rates of >35%, the proposed follow-up study is adequately powered for the expected size of effect for *Climate Substance Use* (*d*=0.15), *Climate Mental Health* (*d*=0.15), and the *CSC* intervention (*d*=0.2). Power calculations based on the obtained sample, where there were at least 16 schools, and an average of at least 80 students per school in each intervention group show that the power to detect an effect size of *d*=0.15 at the final long-term follow-up would be >90%.

### Measures

Where possible, measures have remained consistent from the original CSC study to the long-term follow-up study. Some measures have been amended or updated to be age appropriate as participants transition out of school. Details of all included measures in the long-term follow-up study are outlined below.

Demographic data including gender, age, country of birth, truancy rates, and academic performance were obtained at baseline to determine the equivalence of groups. All follow-up outcomes will be assessed by validated self-report measures, which have been shown to be valid and reliable in adolescent populations [53-55].

#### Primary Measures

##### Alcohol Use

Drinking behaviors in the past 6 months will be assessed using an adapted version of the Patterns of Alcohol index [56]. Participants will report the frequency and average quantity of their alcohol consumption in standard drinks, frequency of binge drinking (defined as consuming 5 or more standard drinks on 1 occasion), the maximum number of drinks consumed on 1 occasion, and the proportion of their friends or acquaintances who drink alcohol and drink to get drunk. From this scale, it will be possible to calculate dichotomous variables to determine whether participants have ever had a sip, full serve of alcohol, or binge drunk (consumed 5 or more standard drinks). This questionnaire has been used in previous *Climate Schools* trials [57-59] and allows for comparison with large-scale Australian cohorts. A standard drinks chart will be presented with these items to assist reporting. Emerging symptoms of alcohol use disorder will be screened for by a Diagnostic and Statistical Manual of Mental Disorders-fifth edition (DSM-5) symptom checklist [60]. This 16-item checklist examines the presence of symptoms in the past 12 months as specified in the DSM-5 and includes items such as, “during the past 12 months, have you tried unsuccessfully to reduce your use of alcohol?” Alcohol-related harms will be measured by the 24-item Brief Young Adult Alcohol Consequences Questionnaire [61]. Items capture a wide range of age-appropriate harms from mild (eg, fatigue) to more severe (eg, sexual victimization) consequences during the past year.

##### Cannabis Use

Cannabis use will be assessed by 4 items from the National Drug and Alcohol Strategy Household Survey (NDSHS) [62]. Items will assess whether participants have ever tried cannabis, their frequency of use in the past 6 months, and proportion of their friends and acquaintances who use cannabis. Emerging symptoms of cannabis use disorder will be screened by a 17-item DSM-5 symptom checklist, which assesses the absence or presence of symptoms in the past 12 months [60].

##### Other Substance Use

A total of 6 items from the NDSHS [62], allowing for comparison with a large representative group of Australians, will ask participants whether they have ever tried amphetamines, ecstasy, hallucinogens, sedatives, inhalants, or any other substance and their frequency of use in the last 6 months (on a 5-point scale ranging from “none” to “more than five times”). Questions will be presented alongside a table with alternative names for drugs. Cigarette and electronic cigarette use will be measured by 7 questions modified from Barrington-Trimis et al [63]. Questions assess whether participants have ever tried cigarettes or electronic cigarettes, their frequency of use in the past 6 months and 30 days, and the average quantity of cigarettes smoked per day in the past 30 days.

##### Mental Health Measures

Psychological distress in the past month will be assessed by the Kessler 6 scale [64] and the Distress Questionnaire-5 [60]. Depressive and anxiety symptoms during the past 2 weeks will be measured by the Patient Health Questionnaire-9 modified for adolescents [65] and the 7-item Generalized Anxiety Disorder scale, respectively [66]. The 3-item Mini Social Phobia Inventory [67] will screen for social phobia, whereas the Community Assessment of Psychic Experiences Positive Scale-15 [68] will measure psychotic-like experiences during the past 3 months. A total of 3 dichotomous questions drawn from the Youth Risk Behavior Surveillance System [69] and 1 question from the Patient Health Questionnaire-9 [65] will assess suicidal ideation in the past 12 months, including thoughts of and plans to attempt suicide.

##### Resource Utilization

Participants’ will be consented for access to Medicare Benefits Schedule and Pharmaceutical Benefits Scheme data providing detailed information on the number and cost of contacts with health care professionals and prescription medications reimbursed through these commonwealth-funded plans. These data will be obtained from the Department of Human Services for up to a 4.5-year period from the date of extraction. A retrospective 12-month questionnaire will also be used to capture resource use outside of Medicare Benefits Schedule and Pharmaceutical Benefits Scheme data, in addition to capturing some overlapping data for those participants who may not agree to this data access. The resource use questionnaire was adapted from the Client Services Receipt Inventory [70] and will assess the frequency of contact with health care professionals, other service utilization (eg, ambulance, self-help materials), overnight medical admissions, use of prescription medications, time off paid and unpaid work, support payments, and living arrangements.

##### Health-Related Quality of Life

Health outcomes will be assessed by the Child Heath Utility-9D [71], a pediatric health-related quality of life measure providing utility values for economic evaluations, which has been adapted for Australians aged 18 to 29 years [72].

#### Secondary Measures

##### Social Networks

All participants completing Web versions of the survey will also complete a social networks survey at each time point consisting of questions adapted from O'Malley et al [73] and Lau-Barraco et al [74]. Participants will be asked to nominate up to 6 people with whom they spent most of their free time with in the past 12 months. For each identified person, participants will be asked to report on this person’s demographics, relationships, frequency and mode of contact, perceived mental health symptoms, perceived alcohol consumption, whether they are considered a drinking associate, and relationship closeness. The relationships and closeness between pairs of nominated individuals will also be rated. Although these questions were not included in the original phase of the study, a subsample of participants were asked to nominate their 6 closest friends in their year at school to provide information on their social networks in the original CSC study.

##### Other Measures

Other secondary measures that will be administered include the following: (1) Bandura’s Resistive Self-Regulatory Efficacy Scale [75,76] will examine perceived self-efficacy to resist peer pressure to engage in high-risk activities; (2) the 8-item Frost Multidimensional Perfectionism Scale-Brief to measure perfectionism across 2 dimensions (striving and evaluative concerns) [77]; (3) the 11-item Emotion Regulation Questionnaire to assess individual differences in the use of 2 emotional regulation strategies (reappraisal and suppression) [78]; and (4) the Strengths and Difficulties Questionnaire 18+ to assess both internalizing and externalizing symptoms of participants [79].

### Statistical Analysis

Intention-to-treat analyses will be carried out for all primary and secondary outcomes in the trial, including all participants in the groups they were initially randomized to. Multilevel mixed-effects regression models will be used to assess these outcomes. Where appropriate, generalized mixed-effects models will be applied, for example, using logistic regression for dichotomous outcomes.

Multilevel models are able to account for the clustered design of the trial by taking into account the expected correlations between the multiple observations of each participant and between participants in the same school [80]. Multilevel models will include random intercepts for schools and random intercepts and slopes for time for individuals. The best fitting random effects structure for each model will be determined using likelihood ratio tests and model fit statistics such as the Akaike information criterion.

Models will include dummy-coded intervention terms that compare each intervention with the reference control group and time terms reflecting the survey occasion, along with covariates such as gender to adjust for possible confounding. The effects of greatest interest for assessing the effectiveness of the interventions are intervention × time terms that provide baseline-adjusted estimates of how each intervention group has changed relative to control. Interpretable measures of effect size such as odds ratios and standardized mean differences will be calculated for all effects as well their accompanying CIs.

Given that some outcome data are expected to be missing due to loss to follow-up, the analysis must also account for missing data. As mixed-effects models employ maximum likelihood estimation, they produce unbiased estimates when missing data can be assumed to be either missing completely at random or missing at random [81] and are considered to be superior to other strategies for dealing with missing data [82].

#### Planned Comparisons

The primary aims of the original CSC trial were to assess the efficacy of the combined CSC intervention in comparison with the stand-alone *Climate Substance Use* intervention, *Climate Mental Health* intervention, and standard education received by the control group [83]. Therefore, planned comparisons for each outcome will compare *CSC versus* Control, *CSC versus Climate Mental Health*, and *CSC versus Climate Substance Use* including all participants allocated to each of these intervention groups.

#### Economic Evaluation

The cost to deliver each intervention will be combined with the additional resources used by participants over the follow-up period to calculate total costs from the Australian health sector and societal perspectives as recommended by current guidelines [84]. Intervention costs will comprise software development, staff, and teacher time to deliver interventions and usual care. Additional health care resources will be valued by applying standard Australian unit costs (ie, Independent Hospital Pricing Authority, Australian Bureau of Statistics wage rates) to the resource use units collected. The combined CSC intervention group will be compared with the stand-alone intervention groups and the control group in terms of both total costs and outcomes as assessed by an incremental cost-effectiveness ratio. Nonparametric bootstrapping will be used to obtain CIs for cost-effectiveness ratios, as parametric techniques are inappropriate for use on skewed variables and ratios. The sensitivity of the results will be tested against the variation in the utility weights and unit cost prices.

#### Additional Analyses: Moderation of Intervention Effects

To explore possible mechanisms for the interventions’ effectiveness, planned moderation analyses will be conducted to examine whether measures of baseline risk moderate the intervention effects. Baseline measures of risk will be investigated in relation to alcohol and other substance use, harms related to substance use, and mental health symptoms.

## Results

The CSC long-term follow-up study is funded from 2018 to 2022 by the Australian National Health and Medical Research Council (APP1143555). The first follow-up wave commences in August 2018, and the results are expected to be submitted for publication in 2022.

## Discussion

### Overall Aim

This paper outlines the study protocol and design of an extended long-term follow-up of the CSC study cohort into late adolescence and early adulthood. The study aims to (1) examine the long-term effectiveness of a combined universal mental health and substance use program (CSC program) in preventing substance use (and related harms) and reducing mental health symptoms up to 7 years post baseline and (2) evaluate the cost-effectiveness of the program over the long term. In addition, we will explore intervention effects on secondary outcomes including self-efficacy, social networks, peer substance use, emotion regulation, and perfectionism into young adulthood as well as key mediators and moderators of intervention effects.

### Strengths and Limitations

This study will address a significant gap in knowledge by determining for the first time the longevity of school-based universal prevention for substance use and mental health delivered via the Web into young adulthood as well as conducting 1 of the first cost-effectiveness studies of Web-based prevention for mental health and substance use up to 7 years post baseline. Furthermore, this will be the first study to examine unique effects of combining substance use and mental health prevention over the long term. As with the original CSC study, 2 key limitations of the study are participant attrition and reliance on self-report for the majority of measures. Although follow-up rates for the original CSC study remained relatively high across survey waves (ranging from 67% to 88%), it is anticipated that the addition of a new round of consent and participants transitioning from school to postschool environments in this study will present additional challenges and increase study attrition. Anticipated barriers include incomplete and changing contact details, participant relocation (overseas or interstate for travel, study, or work opportunities), and a lack of follow-up support from teachers as participants complete school. To aid in participant follow-up, a set of detailed follow-up strategies will be developed, including a procedure using a wide range of mediums to contact participants (email, SMS, Facebook, phone calls, and mail out), obtaining contact details from one other person who is likely to know how to contact the participant should their contact details change, and adequately reimbursing participants for their time (Aus $20 reimbursement). Reliance on self-report data for the majority of collected measures may introduce bias related to social desirability, particularly in relation to illegal or risky behaviors such as drug use. Nonetheless, self-reported substance use has been shown to be both reliable and valid [53,54], especially when confidentiality is assured and when young people self administer surveys on the Web [55,85], both of which will occur in this study.

### Conclusions

Harms relating to early substance use and development of mental health problems are a serious concern, and the transition into early adulthood represents a key risk period. Despite this, very little is currently known about the effectiveness of school-based prevention programs beyond school age. This study addresses a critical knowledge gap and will indicate if prevention approaches for anxiety, depression, and substance use can have lasting effects. Furthermore, this study will provide a critical economic evaluation of the long-term effects of a combined universal approach to prevent substance use and mental health problems among young people. This knowledge is vital to inform policy both nationally and internationally as economic modeling suggests substantial societal benefit can be gained from even modest reductions in substance use and mental health [48,50,86]. Evidence of sustained benefits into adulthood would provide a scalable and easy-to-implement prevention strategy that could be disseminated immediately, at minimal cost, to reduce the considerable harms, burden of disease, injury, and social costs associated with substance use and mental disorders.

## Appendix

Multimedia Appendix 1 Consort diagram of the Climate Schools Combined study.

Multimedia Appendix 2 Completed Climate Schools Combined (CSC) Study assessments and timeline for extended follow-up assessments.

## Abbreviations

CSC: Climate Schools Combined
DSM-5: Diagnostic and Statistical Manual of Mental Disorders-fifth Edition
NDSHS: National Drug and Alcohol Strategy Household Survey
RCT: randomized controlled trial
SMS: short messaging service
