# Correction: Evaluating the Long-Term Effectiveness of School-Based Depression, Anxiety, and Substance Use Prevention Into Young Adulthood: Protocol for the Climate School Combined Study

**DOI:** 10.2196/15391

**Published:** 2019-09-26

**Authors:** Louise Birrell, Nicola C Newton, Tim Slade, Catherine Chapman, Louise Mewton, Nyanda McBride, Leanne Hides, Mary Lou Chatterton, Steve Allsop, Annalise Healy, Marius Mather, Catherine Quinn, Cathrine Mihalopoulos, Maree Teesson

**Affiliations:** 1 National Health and Medical Research Council Centre of Research Excellence in Mental Health and Substance Use National Drug & Alcohol Research Centre University of New South Wales Sydney Australia; 2 National Drug Research Institute Curtin University Perth Australia; 3 Centre for Substance Abuse Research School of Psychology University of Queensland Brisbane Australia; 4 Population Health Strategic Research Centre Deakin University Geelong Australia

During recruitment for the current phase of the study, the authors of “Evaluating the Long-Term Effectiveness of School-Based Depression, Anxiety, and Substance Use Prevention Into Young Adulthood: Protocol for the Climate School Combined Study” (JMIR Res Protoc 2018;7(11):e11372) discovered that the participant database contained 25 duplicate records.

These 25 records represented cases where the same participant had been able to create two separate accounts on the website where participant data was recorded and managed. The correct sample size for the original phase of the study is 6386, rather than 6411, after removing these duplicates.

Accordingly, the following changes have been made:

**Abstract**

- Under “Methods” in the sentence beginning “A cluster randomized controlled trial (the CSC study) was conducted with 6411 participants”, the number of participants has been changed from 6411 to 6386.

**Introduction**

- In the subsection “The Climate School Combined Study: First Randomized Controlled Trial of Simultaneous Universal Prevention for Anxiety, Depression, and Substance Misuse” in the sentence “A total of 71 schools and 6411 students aged 13 to 14 years at baseline participated in the trial”, the number of students has been changed from 6411 to 6386.

**Methods**

- In the subsection “Ethical Approval and Consent to Participate”, the sentence “The study was approved by the University of New South Wales Human Research Ethics Committee, Australia (HC13073)” has been changed to “The study was approved by the University of Sydney Human Research Ethics Committee, Australia (2018/906)”.
- In the subsection “Study Design”, the sentence “The final cohort at baseline consisted of 6411 year 8 students from 71 schools (mean age 13.5 years [SD 0.6], 54.78% 3511/6411) female, 81.25% [5209/6411] born in Australia)” has been changed to “The final cohort at baseline consisted of 6386 year 8 students from 71 schools (mean age 13.5 years [SD 0.6], 54.84% [3502/6386] female, 81.24% [5188/6386] born in Australia).”
- In the subsection “Sample Size Calculations” in the sentence “Participants for this study come from 6411 students from 71 schools recruited to the original CSC study”, 6411 has been corrected to 6386. In the sentence “In our original study, we achieved a total sample size of 6411 students”, 6411 has been corrected to 6386.

**Discussion**

- In the subsection “Strengths and Limitations” in the sentence beginning “Although follow-up rates for the original CSC study remained relatively high across survey waves (ranging from 66% to 88%)”, 66% has been corrected to 67%.

**Multimedia Appendices**

- Both multimedia appendices have been updated to reflect the correct number of participants followed up at each occasion.

The corrections will appear in the online version of the paper on the JMIR website on September 26, 2019, together with the publication of this correction notice. Because this was made after submission to PubMed, PubMed Central, and other full-text repositories, the corrected article also has been resubmitted to those repositories.

## Appendix

Multimedia Appendix 1 Consort diagram of the Climate Schools Combined study.

Multimedia Appendix 2 Completed Climate Schools Combined (CSC) Study assessments and timeline for extended follow-up assessments.

