# Supplementary figures and images for: Evaluating the Long-Term Effectiveness of School-Based Depression, Anxiety, and Substance Use Prevention Into Young Adulthood: Protocol for the Climate School Combined Study
Source: JMIR Res Protoc. 2018 Nov 6;7(11):e11372. doi: 10.2196/11372 (PMC6246975; doi:10.2196/11372)

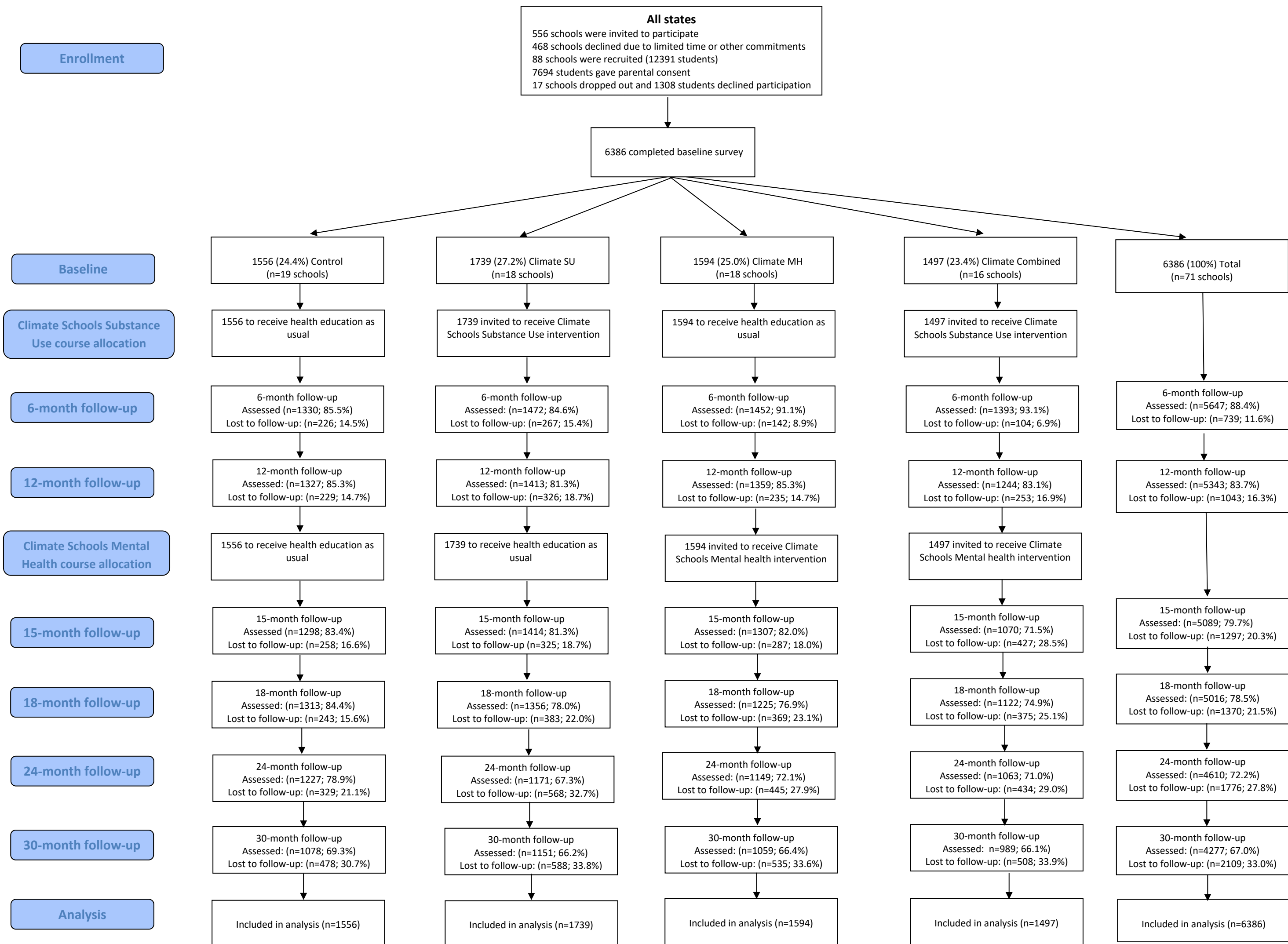

Supplement: Multimedia Appendix 1 [file resprot_v7i11e11372_app1.pdf]
