# Supplementary material for: Evaluating the Long-Term Effectiveness of School-Based Depression, Anxiety, and Substance Use Prevention Into Young Adulthood: Protocol for the Climate School Combined Study
Source: JMIR Res Protoc. 2018 Nov 6;7(11):e11372. doi: 10.2196/11372 (PMC6246975; doi:10.2196/11372)
Supplement: Multimedia Appendix 2 [file resprot_v7i11e11372_app2.pdf]

Original CSC trial[a]										Extended follow-up		
2014 Term 1	2014 Term 1	2014 Term 3	2014 Term 3	2015 Term 1	2015 Term 1	2015 Term 2	2015 Term 3	2016 Term 1	2016 Term 3	August 2018 -June 2019	January 2020 -June 2020	January 2021 -June 2021
13.5 years old	Substance use (module 1)	Substance use (module 2)	14 years old	14.5 years old	Mental health module	14.75 years old	15 years old	15.5 years old	16 years old	18.5 years old	19.5 years old	20.5 years old
Baseline			6-month F/U	12-month F/U		15-month F/U	18-month F/U	24-month F/U	30-month F/U	5-year F/U	6-year F/U	7-year F/U
n=6386			n=5647	n=5343		n=5089	n=5016	n=4610	n=4277	↔[b]	↔	↔
Follow-up rate			(88.43%)	(83.67%)		(79.69%)	(78.55%)	(72.19%)	(66.97%)			

[a]Note. Participants in the state of Queensland (QLD) complete school 1 year earlier than participants in New South Wales (NSW) and Western Australia (WA). To collect data from QLD participants in their first year post school, follow-up will commence in QLD from August to January, whereas data collection will run from January to June in NSW and WA.

[b]Symbol (↔) indicates that data has not yet been collected.
